# Central corneal thickness changes in bevel-up versus bevel-down phacoemulsification cataract surgery: study protocol for a randomised, triple-blind, parallel group trial

**DOI:** 10.1136/bmjopen-2016-012024

**Published:** 2016-09-29

**Authors:** Soujanya Kaup, Siddharudha Shivalli, Divyalakshmi KS, Cynthia Arunachalam, Rejitha Chinnu Varghese

**Affiliations:** 1 Department of Ophthalmology, Yenepoya Medical College, Yenepoya University, Mangalore, Karnataka, India; 2 Department of Community Medicine, Yenepoya Medical College, Yenepoya University, Mangalore, Karnataka, India

**Keywords:** central corneal thickness, endothelial damage, phaco tip position, phacoemulsification

## Abstract

**Introduction:**

Corneal endothelial damage following phacoemulsification is still one of the major concerns of modern day cataract surgery. Although many techniques have been proposed, the risks of posterior capsular rupture and corneal endothelium damage persist. In theory, damage to the corneal endothelium is minimised by delivering the lowest phaco energy only in the direction necessary to emulsify the lens nucleus. Hence, it is believed that the bevel of the needle should be turned towards the nucleus or the nuclear fragment (ie, bevel-down. However, there is a difference of opinion among ophthalmologists with reference to the phaco tip's position (bevel-up vs bevel-down) during phacoemulsification. This subject has not been extensively studied earlier.

**Methods and analysis:**

This is a prospective, triple-blinded (trial participant, outcome assessor and the data analyst), randomised controlled trial with 2 parallel groups and with an allocation ratio of 1:1. It will be conducted in a tertiary care hospital, Mangaluru, India. The objective is to compare the postoperative central corneal thickness changes between the bevel-up and bevel-down techniques of phacoemulsification. Patients aged >18 years with immature cataract undergoing phacoemulsification will be selected for the study. The important exclusion criteria are the history of previous significant ocular trauma or intraocular surgery, corneal pathology, pseudoexfoliation syndrome, intraocular inflammation, a preoperative fully dilated pupil <6 mm, anterior chamber depth <2.5 mm and nuclear sclerosis grade >4. After randomisation, patients will undergo phacoemulsification surgery either by a bevel-up or bevel-down procedure. With an estimated power of 80%, the calculated sample size is 55 patients in each group. The recruitment will start from April 2016.

**Ethics and dissemination:**

Yenepoya University Ethics Committee, India has approved the study protocol (YUEC/148/2016 on 18 February 2016). It complies with the Declaration of Helsinki, local laws and the International Council for Harmonization-good clinical practices.

**Trial registration number:**

CTRI/2016/02/006691; Pre-results.

**Strengths and limitations of this study**

- This study attempts to find the most cornea-friendly technique of clear corneal phacoemulsification with regard to phaco tip's position.
- Study findings would be more pertinent to those who are at high risk of corneal decompensation (ie, small pupil, short anterior chamber depth, high nuclear grade, large nucleus, older age, etc) following phacoemulsification.
- Multiple factors can affect the study outcome (central corneal thickness).

## Introduction

Corneal endothelial damage following phacoemulsification is still one of the major concerns of modern day cataract surgery. Introduction of different techniques and certain innovations in the phaco instrumentation have reduced the incidence of postoperative corneal endothelial loss. At every stage of cataract surgery, the surgeon has options to optimise corneal endothelial safety. This includes relevant parameters in various phaco platforms, surgical techniques, materials used and patient characteristics such as the grade of the cataract, age of the patient and the presence of corneal disease.

Several mechanisms have been proposed for endothelial cell damage during phacoemulsification; these include mechanical contact with nuclear fragments, irrigation flow, turbulence and movement of fluid, direct trauma caused by instruments or lens fragments, and formation of cavitation bubbles.^1^ ^2^

In phacoemulsification procedures the most important predictor of corneal clarity in the early postoperative period is inversely proportional to the amount of ultrasound energy used. For this reason, all of the major platforms now use power modulation to reduce the amount of phaco power necessary, either by varying the duty cycle and pulse energy or by varying the pulse intervals with microburst techniques.

Several phacoemulsification techniques have been developed and modified.^3–7^ Although many techniques have been proposed, the risks of posterior capsular rupture and corneal endothelium damage persist. In theory, damage to the corneal endothelium is minimised by delivering the lowest phaco energy only in the direction necessary to emulsify the lens nucleus. Furthermore, phacoemulsification should occur in the posterior chamber rather than in the iris plane or the anterior chamber.^8^ Hence, it has traditionally been believed that the bevel of the needle should be turned towards the nucleus or the nuclear fragment (ie, bevel-down).^9^ However, Faramarzi *et al*^10^ hypothesise that in the bevel-up technique, the phaco tip, and therefore the source of heat, are farther from the endothelial cells than in the bevel-down technique and this decreases the chance of endothelial cell damage. Further another clinical trial by Raskin *et al*^11^ concluded in their study that bevel-up tip position has a negative effect on corneal endothelial cells compared with the bevel-down position. Hence, there is a difference of opinion among ophthalmologists with respect to the phaco tip position during phacoemulsification. This subject has not been extensively studied earlier, hence this study is planned.

## Objective

To compare the postoperative central corneal thickness (CCT) changes between bevel-up and bevel-down techniques of phacoemulsification.

*Trail design*: Prospective randomised, parallel group, exploratory, triple-blind (trial participant, outcome assessor and the data analyst) trial with an allocation ratio of 1:1.

*Trial registration*: This trial is registered prospectively in the Clinical trial registry of India (CTRI/2016/02/006691) and the full trial protocol can be accessed on http://ctri.nic.in/Clinicaltrials/showallp.php?mid1=14144&EncHid=&userName=CTRI/2016/02/006691.

## Methods

*Ethics and dissemination*: Written informed consent will be taken from all study participants. It complies with the Declaration of Helsinki, local laws and the International Council for Harmonisation-good clinical practices (ICH-GCP). After obtaining the written informed consent (by SK), protected personally identifiable information (PPII) will be replaced by the research identification code. Face sheets containing PPIIs will be removed from the completed questionnaire. Access to master code list will be limited to SK and DKS. Research data will be stored securely in locked cabinets and the relevant electronic data will be stored in the password-protected computers and files. The intervention in this trial is the standard treatment for the eligible participants and does not pose an additional risk. Hence, the trial participants will not be eligible for any damage compensation. Following analysis of the data, the results will be presented in scientific forums and published in scientific journals.

*Study setting*: Department of Ophthalmology, Yenepoya Medical College Hospital, Yenepoya University, Mangaluru, India.

*Study period*: January 2016 to December 2017.

### Participants and methods

*Inclusion criteria*: Consecutive patients aged >18 years with immature cataract undergoing phacoemulsification at Yenepoya Medical College Hospital, Mangaluru, India will be selected for the study.

*Exclusion criteria*: History of previous significant ocular trauma or intraocular surgery, corneal pathology, pseudoexfoliation syndrome, intraocular inflammation, a preoperative fully dilated pupil smaller than 6 mm, anterior chamber depth (ACD) <2.5 mm and nuclear sclerosis grade >4. Cases with intraoperative complications, those where direct chop could not be done and patients not willing to participate in the study will be excluded.

### Interventions (bevel-up and bevel-down phacoemulsification groups)

Patients will be randomised into two groups, that is, bevel-up and bevel-down. The phaco tip will be held in the bevel-down position for the bevel-down group and in bevel-up position for the bevel-up group whenever the phaco tip is introduced into the anterior chamber. The preoperative evaluation, rest of the surgical procedure, phaco parameters and intraoperative and postoperative evaluations will remain the same in both the groups. All the surgeries will be performed by a single surgeon (SK).

*Preoperative evaluation*: In all the cases, a complete preoperative ocular examination will be performed including uncorrected distance visual acuity (UDVA), corrected distance visual acuity (CDVA), slit lamp evaluation, applanation tonometry, posterior pole evaluation with a non-contact 78 dioptre lens and indirect ophthalmoscopy. The axial length (AL) and ACD will be measured by using an ultrasonic A-scan (Echorule Pro, Biomedix Optotechnik & Devices). CCT will be measured using an ultrasound pachymeter (Pacscan 300P, Ver 3 Rev U, Sonomed). The mean of 5 CCT readings with an SD ≤0.09, with the patient fixating at a target, will be recorded. Nuclear hardness will be evaluated clinically with slit lamp biomicroscopy according to the colour of the nucleus based on the Lens Opacities Classification System III.^12^

### Surgical procedure

Two limbal stab incisions will be made. The anterior capsule will be stained with trypan blue dye after which, the anterior chamber will be filled with hydroxypropyl methylcellulose 2%. A capsulorhexis 5.0–5.5 mm in diameter will be created with a needle cystotome. A 2.8 mm clear corneal incision will be made, followed by hydrodissection and hydrodelineation of the nucleus.

Phacoemulsification of the nucleus will be performed using the phaco chop technique with a 30° 19-gauge phaco tip. In both groups, the nucleus will be stabilised with the phacoemulsification tip, which is impaled with moderate vacuum and low ultrasonic power. The chopper will be advanced under the anterior capsule until it can pass around the equator of the nucleus at the nucleus–epinucleus border, 180° from the phaco tip. The chopper will be advanced towards the phaco tip splitting the nucleus into half. By rotating the lens, several pieces of nucleus are broken (chopped). The pieces will then be emulsified. Following this, irrigation/aspiration of cortical material will be performed with a bimanual irrigation/aspiration cannula. Next, a foldable hydrophilic acrylic intraocular lens with ultraviolet absorbing optic and square edge design (Acryfold, Appasamy ocular devices, Pondicherry, India) will be implanted in the capsular bag using a disposable injector cartridge system. Finally, the anterior chamber will be irrigated, the ocular viscoelastic device will be removed, the wounds will be secured by stromal hydration and the eye will be patched.

#### Phaco parameters

All the operations will be performed with Sovereign compact phacoemulsification system with WhiteStar technology and Ellips (Abbott Medical Optics, Abbott Laboratories, Abbott Park, Illinois, USA). During direct chop, the parameters will be kept as follows: maximum aspiration flow rate: 28 cc/min, maximum vacuum: 295 mm Hg and threshold vacuum: 125 mm Hg; maximum power: 30 continuous 6/12 (33%) with Whitestar on and occluded 40 linear long pulse 6/12 (33%) with Whitestar on. The parameters for higher grades of cataract will be as follows: maximum aspiration flow rate: 28 cc/min, maximum vacuum: 400 mm Hg and threshold vacuum 250 mm Hg; maximum power: 40 continuous 6/12 (33%) with Whitestar on and occluded 40 linear long pulse 6/12 (33%) with Whitestar on.

### Intraoperative evaluation

The parameters to be evaluated intraoperatively include mean phaco power (%), effective phaco time (EPT) (seconds) and phaco time using Ellips FX technology (EFX). The EFX is roughly the EPT with a specific coefficient for the transversal movement of the phaco tip expressed in seconds. Amount of irrigating fluid used will also be noted.

### Postoperative evaluation

All the patients will be put on topical ciprofloxacin and dexamethasone eye drops combination, one drop six times a day for 1 week for the first week, after which it will be gradually tapered over a period of 1 month. All the patients will be examined postoperatively on day 1, day 7 and at the end of 1 month. The examinations will include UDVA, CDVA, slit lamp biomicroscopy, applanation tonometry, funduscopy and CCT measurement. Telephonic reminders will be made to encourage the complete follow-up.

*Outcome measure*: The CCT will be measured on days 1, 7 and 30. Deturgescence of the corneal stroma is controlled by the pumping action of the endothelial layer and can be monitored by the measurement of CCT. Loss or damage of endothelial cells leads to an increase in corneal thickness, which may ultimately induce corneal decompensation and loss of vision.^13^ A significant linear correlation exists between increase in corneal thickness in the immediate postoperative period and percentage of endothelial cell loss.^14^ Hence, CCT measurement can be used as a simple tool to assess the amount of corneal endothelial damage postcataract surgery.

**Figure 1:**
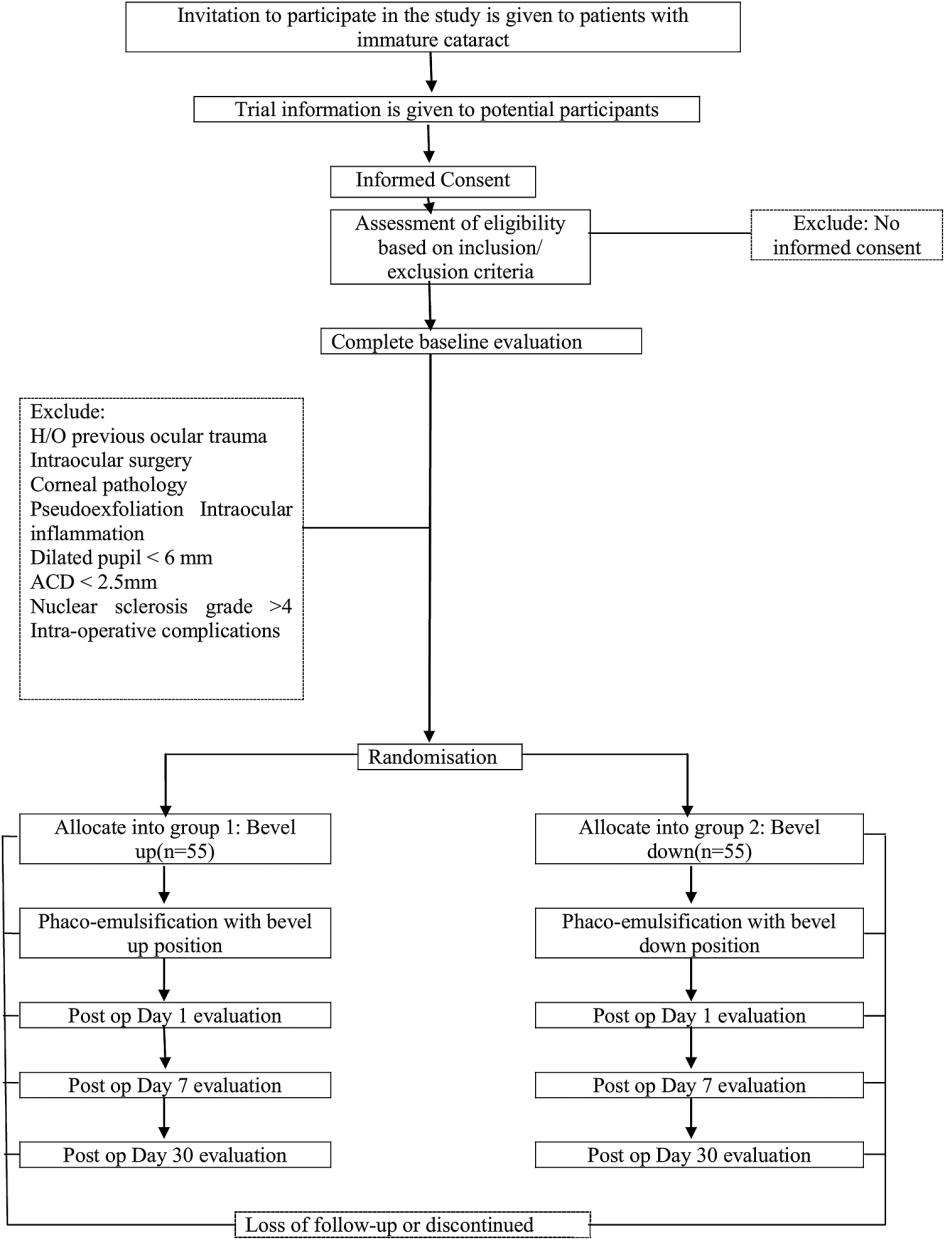
Flow diagram of the trial participants. ACD, anterior chamber depth.

Figure 1 shows the proposed flow of the participants in this trial.

*Sample size calculation*: The sample size calculation is based on the anticipated differences in the CCT (mean±SD) between the two groups. A sample of 50 in each group is needed to detect a mean CCT difference of 20 µm with an SD of 35.3,^15^ 1:1 allocation ratio, α error of 0.05 and a power of 80%.^16^ The final sample is inflated to 55 with due consideration of 10% attrition.

*Sensitivity analysis of sample size calculation for CCT*: The power of the study will be estimated based on the actual number of the study participants who complete the study and the observed differences in CCT across the two study groups.

#### Randomisation and blinding

SS will generate the random sequence by computer-generated random numbers and it will be secured in sealed envelopes. SK will perform the surgeries and RCV will assess the outcome. The trial participants, outcome assessor and data analyst will be blinded.

## Data collection and statistical analysis

A detailed statistical analysis plan will be prepared. A blind review of the data will be performed after the end of the planned follow-up period without looking at the randomised treatment for each trial participant. Attritions will be excluded from the analysis. Intention-to-treat principle will be used for the analysis. Results will be expressed in proportion and means (±SD) for categorical and continuous variables, respectively. Student's t-test will be used to compare CCTs between the study groups. A repeated measures analysis of variance will be conducted to assess for any difference in CCT between bevel-up and bevel-down groups over time. All the data will be analysed using SPSS V.16.

### Data monitoring committee

An independent data monitoring committee (DMC) will be established. The DMC will monitor the course of the trial and if necessary will give a recommendation to the main investigator of the trial for discontinuation, modification or continuation of the study and the same shall be communicated to the University's Ethics Committee and Clinical Trial Registry of India.

### Safety parameters

All the adverse events will be listed and displayed in summary tables. The total number of adverse events, the minimum, maximum and mean number of adverse events per patient will be reported.

### Study progress

The study began in January 2016, ethical approval and trial registration number were obtained in February 2016. The recruitment will begin in April 2016.

## Discussion

The importance of preserving the corneal endothelium during intraocular surgeries has been well established. Postoperative corneal decompensation is rare when, modern phaco technology is used in combination with proper surgical techniques. However, there are several patient-based factors that increase the likelihood of corneal decompensation. With patients electing to have cataract surgery and refractive lens exchange at increasingly younger ages, protecting the endothelial cell layer at all times is essential to avoid the potential for an epidemic of pseudophakic bullous keratopathy in the future.^17^ Patient expectations after cataract surgery continue to increase, and the patients anticipate perfect vision from as early as the first postoperative day.^17^ The main determinant of a patient's visual acuity on postoperative day 1 is the extent to which the endothelium has been protected.^18^ Hence, one must strive to achieve a surgical technique which causes the least damage to the corneal endothelium. This becomes even more imperative while operating on patients who are prone to have a higher endothelial cell loss during phacoemulsification. These include patients with shorter AL, small pupil, short ACD, high nuclear grade, large nucleus, older age, etc.^19–21^ Hence, in this study an attempt will be made to determine the effect of altering the position of phaco tip (bevel-up vs bevel-down) on CCT.

## References

[1] Linebarger EJ, Hardten DR, Shah GK Phacoemulsification and modern cataract surgery. Surv Ophthalmol 1999;44:123–47. 10541151 10.1016/s0039-6257(99)00085-5

[2] Kim EK, Cristol SM, Geroski DH Corneal endothelial damage by air bubbles during phacoemulsification. Arch Ophthalmol 1997;115:81–8. 9006430 10.1001/archopht.1997.01100150083014

[3] Kelman CD Phaco-emulsification and aspiration; a new technique of cataract removal: a preliminary report. Am J Ophthalmol 1967;64:23–35. 6028631

[4] Gimbel HV Divide and conquer nucleofractis phacoemulsification: development and variations. J Cataract Refract Surg 1991;17:281–91. 1861242 10.1016/s0886-3350(13)80824-3

[5] Shepherd JR In situ fracture. J Cataract Refract Surg 1990;16:436–40. 2380923 10.1016/s0886-3350(13)80796-1

[6] Fine IH, Maloney WF, Dillman DM Crack and flip phacoemulsification technique. J Cataract Refract Surg 1993;19:797–802. 8271182 10.1016/s0886-3350(13)80355-0

[7] Koch PS, Katzen LE Stop and chop phacoemulsification. J Cataract Refract Surg 1994;20:566–70. 7996415 10.1016/s0886-3350(13)80239-8

[8] Davison JA Minimal lift-multiple rotation technique for capsular bag phacoemulsification and intraocular lens fixation. J Cataract Refract Surg 1988;14:25–34. 3339544 10.1016/s0886-3350(88)80060-9

[9] Steinert RF Cataract surgery. Elsevier Health Sciences, 2010:64.

[10] Faramarzi A, Javadi MA, Karimian F Corneal endothelial cell loss during phacoemulsification: bevel-up versus bevel-down phaco tip. J Cataract Refract Surg 2011;37:1971–6. 10.1016/j.jcrs.2011.05.034 21940143

[11] Raskin E, Paula JS, Cruz AAV Effect of bevel position on the corneal endothelium after phacoemulsification. Arq Bras Oftalmol 2010;73:508–10. 21271025 10.1590/s0004-27492010000600008

[12] Chylack LT Jr, Wolfe JK, Singer DM The lens opacities classification system III. Arch Ophthalmol 1993;111:831–6. 8512486 10.1001/archopht.1993.01090060119035

[13] Ventura AC, Wälti R, Böhnke M Corneal thickness and endothelial density before and after cataract surgery. Br J Ophthalmol 2001;85:18–20. 11133705 10.1136/bjo.85.1.18 PMC1723680

[14] Cheng H, Bates AK, Wood L Positive correlation of corneal thickness and endothelial cell loss. Serial measurements after cataract surgery. Arch Ophthalmol 1988;106:920–2. 3390055 10.1001/archopht.1988.01060140066026

[15] Vijaya L, George R, Arvind H Central corneal thickness in adult South Indians: the Chennai Glaucoma Study. Ophthalmology 2010;117:700–4. 10.1016/j.ophtha.2009.09.025 20079536

[16] Dhand NK, Khatkar MS Statulator: An online statistical calculator. Sample Size Calculator for Comparing Two Independent Means (cited 8 March 2016). http://statulator.com/SampleSize/ss2M.html

[17] Barsam A. Protecting the Endothelium During Phacoemulsification [Internet]. CRSTEurope. http://crstodayeurope.com/articles/2015-apr/protecting-the-endothelium-during-phacoemulsification/ (accessed 9 Mar 2016).

[18] Donnenfeld ED, Holland EJ, Solomon KD A multicenter randomized controlled fellow eye trial of pulse-dosed difluprednate 0.05% versus prednisolone acetate 1% in cataract surgery. Am J Ophthalmol 2011;152:609–17.e1. 10.1016/j.ajo.2011.03.018 21704965

[19] Hayashi K, Hayashi H, Nakao F Risk factors for corneal endothelial injury during phacoemulsification. J Cataract Refract Surg 1996;22:1079–84. 8915805 10.1016/s0886-3350(96)80121-0

[20] Walkow T, Anders N, Klebe S Endothelial cell loss after phacoemulsification: relation to preoperative and intraoperative parameters. J Cataract Refract Surg 2000;26:727–32. 10831904 10.1016/s0886-3350(99)00462-9

[21] Hwang HB, Lyu B, Yim HB Endothelial cell loss after phacoemulsification according to different anterior chamber depths. J Ophthalmol 2015;2015:210716 10.1155/2015/210716 26417452 PMC4568363

